# A Stratified Precision Medicine Trial Targeting Selective Mechanisms of Alpha 2A Agonism as a Treatment for the Cognitive Biotype of Depression: The BIomarker Guided (BIG) Study for Depression

**DOI:** 10.21203/rs.3.rs-5762756/v1

**Published:** 2025-02-27

**Authors:** Leanne Williams, Laura Hack, Jenna Jubeir, Rachel Hilton, Leonardo Tozzi, Leyla Boyar, Xue Zhang, Timothy Lyons, Booil Jo, Ruth O’Hara, Alan Schatzberg

**Affiliations:** Stanford University School of Medicine; Department of Psychiatry and Behavioral Sciences, Stanford University School of Medicine Stanford, CA 94305, USA; Department of Psychiatry and Behavioral Sciences, Stanford University School of Medicine Stanford, CA 94305, USA; Stanford University; Department of Psychiatry and Behavioral Sciences, Stanford University School of Medicine Stanford, CA 94305, USA; Department of Psychiatry and Behavioral Sciences, Stanford University School of Medicine Stanford, CA 94305, USA; Department of Psychiatry and Behavioral Sciences, Stanford University School of Medicine Stanford, CA 94305, USA; Department of Psychiatry and Behavioral Sciences, Stanford University School of Medicine Stanford, CA 94305, USA; Stanford University; Department of Psychiatry and Behavioral Sciences, Stanford University School of Medicine Stanford, CA 94305, USA; Stanford University

## Abstract

Cognitive impairments contribute significantly to psychosocial dysfunction in major depressive disorder (MDD), yet mechanistically selective treatments targeted to these impairments are lacking. We evaluated guanfacine immediate release (GIR), an alpha 2A receptor agonist, as a novel treatment for selectively improving cognitive control circuit function and behavioral performance in a subtype of depression, the cognitive biotype. Seventeen MDD participants of this biotype completed 6–8 weeks of GIR treatment (target dose: 2mg/night), meeting our per protocol criteria. GIR significantly increased activation and connectivity within the cognitive control circuit. The clinical response rate was 76.5% (defined by ≥ 50% improvement on the 17-item Hamilton Rating Scale for Depression (HRSD-17), exceeding conventional antidepressant rates, and 64.7% achieved remission (HRSD-17 score of ≤ 7). GIR significantly improved cognitive control performance, quality of life, and global life satisfaction. This study is the first to demonstrate both efficacy and target engagement of GIR as a mechanistically selective treatment specifically for the cognitive biotype of depression.

## Main

As many as one-third of patients with major depressive disorder (MDD) do not fully recover after trying multiple antidepressants^1,2^. The outcomes are grave for these unremitted patients; they often become chronically disabled, experience poor quality of life, and are at heightened risk of suicide^3,4^. Even for those who do remit, residual symptoms—which commonly include cognitive dysfunction—can be debilitating^5^. In this study, we focus on cognitive impairment in depression, a leading contributor to non-response to antidepressants^6,7^, poor functional and social outcomes, and increased risk of suicide^8^. Our previous research identified a distinct subgroup, termed “cognitive biotype+”, within the population of adults with depression^9^. This biotype, comprising that 27% of patients, is characterized by pre-treatment global cognitive impairments, particularly in cognitive control domains.

Compared to other individuals with depression, those in the cognitive biotype + subgroup exhibit significantly reduced task-evoked activation and functional connectivity in key regions within the cognitive control circuit, specifically the dorsolateral prefrontal cortex (dLPFC) and dorsal anterior cingulate cortex (dACC). Moreover, they demonstrate a poorer response to standard pharmacotherapy. Based on these findings, we hypothesize that individuals within this cognitive biotype of depression require targeted treatments that address dysfunction within cognitive control circuitry.

The standard drug development pipeline in psychiatry has yielded only one FDA-approved therapy that addresses cognitive deficits in depression: vortioxetine^10^. The precise mechanism by which this multimodal drug improves cognitive function remains unknown. Vortioxetine inhibits the serotonin reuptake transporter, directly modulates multiple serotonin receptors (acting as a partial agonist at 5-HT1B receptors, full agonist at 5-HT1A receptors, and antagonist at 5-HT3, 5-HT1D, and 5-HT7 receptors), and indirectly modulates dopaminergic, noradrenergic, histaminergic, cholinergic, GABAergic, and glutamatergic systems^11^. Multitarget drugs like vortioxetine are typically tested within the standard drug development framework using heterogeneous samples and broad clinical outcomes endpoints. This approach has often failed to produce treatments effective for specific patient subgroups. In the current trial, we take inspiration from Krystal and colleagues’ landmark study, which applied a precision medicine approach, in which the primary outcome was a neural circuit target aligned with both the drug’s mechanism of action and the patient subgroup of interest^12^. In this study, the kappa opioid antagonist Aticaprant produced a measurable change in reward circuit activation as assessed by functional magnetic resonance imaging (fMRI), and improved anhedonia in patients stratified by this symptom dimension of depression.

In the BIomarker Guided (BIG) Study for Depression we applied the same stratified precision medicine approach to the cognitive biotype of depression, using cognitive control circuit activation as our primary neural circuit target assessed by fMRI. This target aligns with the mechanisms of action with the drug we chose to address cognitive impairment, guanfacine immediate release (GIR)^13^. GIR is an alpha 2A (α_2A_) receptor agonist that embodies all the critical components needed to utilize it in the proposed stratified precision medicine study. GIR is FDA-approved for the management of hypertension and used off-label for other disorders. It meets all our criteria for established safety and selectivity, preclinical evidence and established translation from animals to humans. The selectivity of α_2A_ receptors in the prefrontal regions of interest and the high affinity binding of GIR in these regions has been demonstrated using a selective radioligand^17^. Preclinical evidence demonstrates the effect of GIR on increasing activation and plasticity in the prefrontal regions defining the cognitive control circuit^14,15^, an effect that translates across species^13^. An antidepressant-like effect of GIR has also been observed in preclinical work^16^. Human neuroimaging evidence has demonstrated that signal doses of GIR increase dLPFC activation in healthy subjects^17,18^, and in cross-diagnostic samples of patients with impaired cognitive control^19–21^. While we considered the extended-release formulation (GXR), as it is FDA-approved for treating attention-deficit/hyperactivation disorder (ADHD) in children and adolescents, we decided against this option because it has not been studied as extensively for cognitive deficits in adults as has GIR and, to our knowledge, no neuroimaging studies have used this formulation.

We tested the hypotheses that GIR would induce an improvement in cognitive control circuit function, and an improvement in corresponding cognitive behavioral performance. We further hypothesized that GIR would improve depression symptom severity, quality of life, and suicidality. The primary outcome measure was change in activation and connectivity of the cognitive control circuit as defined by cognitive control task-evoked activation in the right and left dLPFC and dACC from pre-treatment baseline to post-treatment. Secondary outcomes included change in depressive symptoms, cognitive control performance, functional capacity, global life satisfaction, and suicidality within the cognitive biotype. To test the association between significant mechanistic primary outcomes and secondary clinical behavioral outcomes, we assessed the repeated measures correlations between cognitive control circuit function and both symptoms and cognitive control performance.

## Results

**Figure 1:**
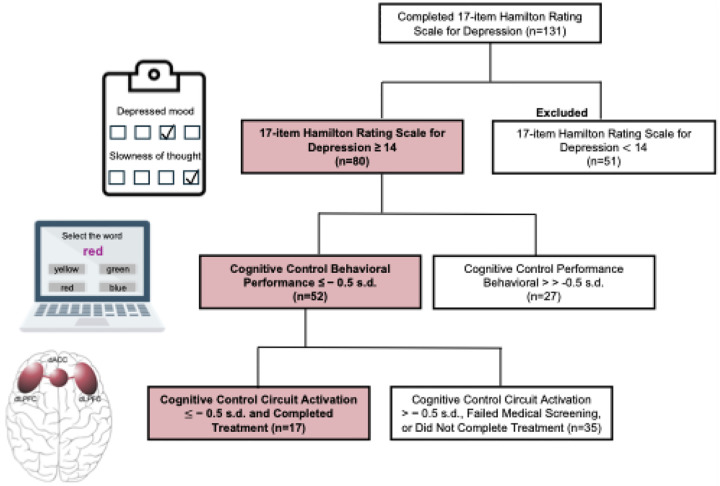
Criteria for defining the cognitive biotype+ subgroup of major depressive disorder. The cognitive biotype+ subgroup of depression was defined using symptom, cognitive control behavioral, and brain measures. Specifically, the following measures and cutoffs were utilized: depression severity assessed by the 17-item Hamilton Rating Scale for Depression of ≥ 14 (represented by clipboard icon); cognitive control behavioral performance assessed by the Maze, Digit Span, and/or the Verbal Interference (Stroop, represented by icon) tasks of ≤ −0.5 s.d. below the normative mean; and GoNoGo task-evoked activation in the left or right dLPFC (regions shown in brain icon) measured by functional magnetic resonance imaging ≤ −0.5 s.d. below the normative mean. Figure excludes interim steps shown in the CONSORT Diagram (Supplementary Fig. 2). *Abbreviations:* dACC = dorsal anterior cingulate cortex; dLPFC = dorsolateral prefrontal cortex; s.d. = standard deviation.

The cognitive biotype + subgroup was defined by depressive symptoms, cognitive control circuit function, and cognitive control performance, and seventeen participants within this biotype completed treatment with 6–8 weeks of GIR (Fig. 1). GIR had a significant effect on each of our primary and secondary outcomes, across circuit, behavior, and symptom modalities.

### Depression symptom outcomes

**Figure 2:**
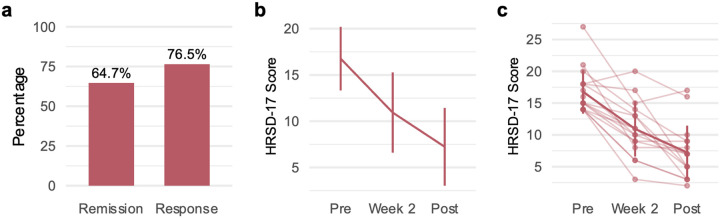
Improvements in depression symptoms following 8 weeks of GIR. Remission and response rates were defined by the HRSD-17. Remission was defined as a HRSD-17 score ≤7, and response was defined as a reduction of ≥50% from baseline. **a**,Rates of remission and response were 64.7% and 76.5%, respectively. **b**,GIR improved depressive symptoms after two weeks (*t*(15)=5.878, *P*<0.001,*d*=1.470, 95% CI=[0.937; 2.002]) and after 8 weeks (*t*(*16*)=12.996, *P*<0.001, Cohen’s *d*=3.152, 95% confidence interval (CI)=[2.757; 3.547]). **c**, Data for individual patients, indicated by individual data points and connected by faint lines with a bolded line for sample mean. Error bars represent standard deviation. All statistical tests were two-sided and not adjusted for multiple comparisons. *Abbreviations:* HRSD-17 = 17-item Hamilton Rating Scale for Depression; GIR = guanfacine immediate release.

As shown in Fig. 2b and c, we found a significant reduction in a continuous measure of HRSD-17 depressive symptoms with large effect sizes for pre- to post-treatment (*t*(16) = 12.996, *P* < 0.001, Cohen’s *d* = 3.152, 95% confidence interval (CI)=[2.757; 3.547]) and pre-treatment to week 2 (*t*(15) = 5.878, *P* < 0.001, *d* = 1.470, 95% CI=[0.937; 2.002]). The response rate following 8 weeks of treatment with GIR was 76.5% (13/17) defined as a ≥ 50% improvement in HRSD-17 score from baseline (Fig. 2a). Of these responders 64.7% (11/17) achieved remission, defined by a HRSD-17 score of ≤ 7.

#### Cognitive control circuit outcomes

**Figure 3:**
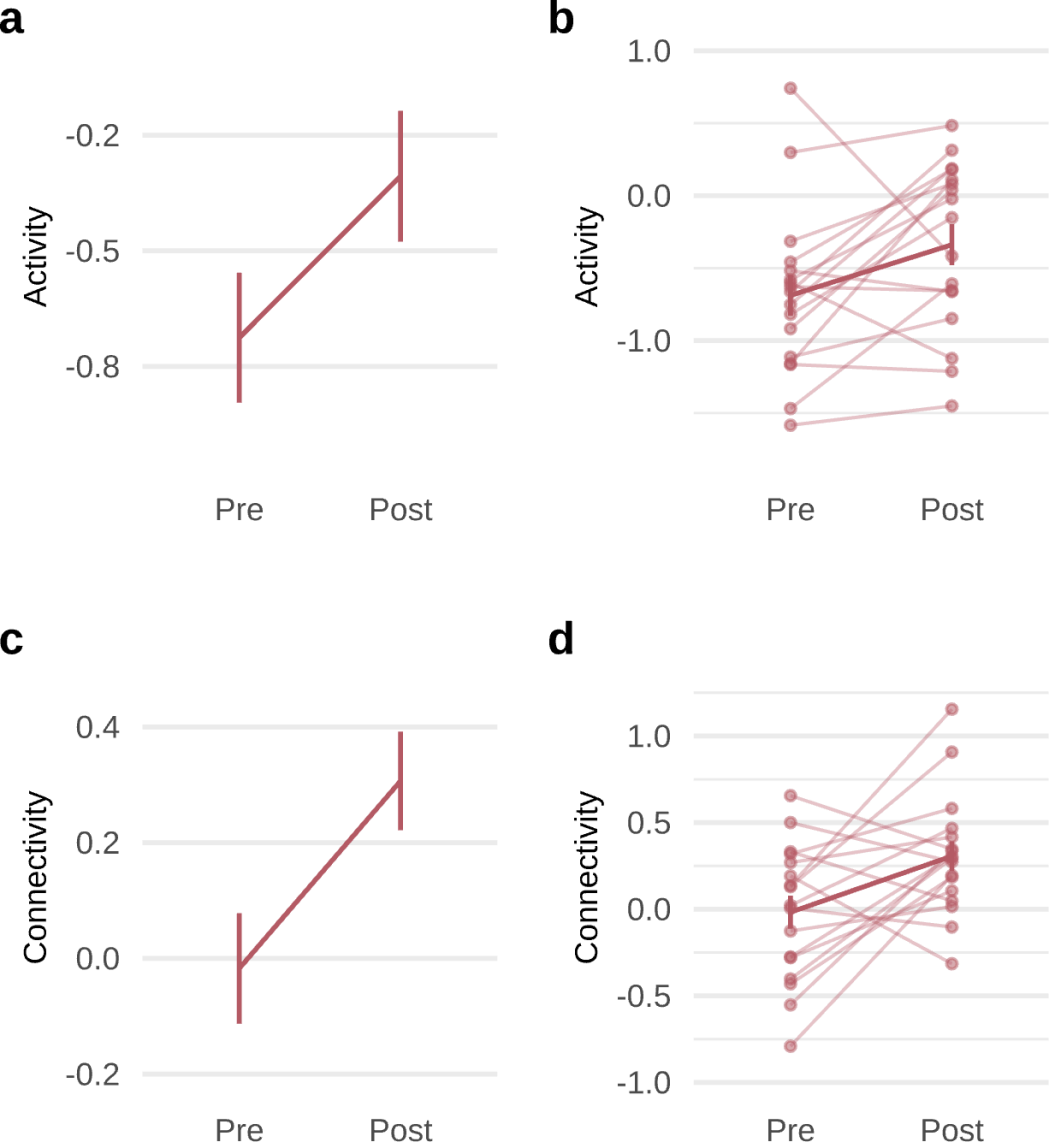
Changes in task-evoked dACC activation and dACC-left dLPFC connectivity following 8 weeks of GIR. **a**, Task-evoked dACC activation increased from pre-treatment to post-treatment (*t*(16)=2.334, *P*=0.033, *d*=0.566, 95% CI=[0.021; 1.112]). **b**, Task-evoked dACC activation data for individual patients, indicated by individual data points and connected by faint lines with a bolded line for sample mean. **c**, Task-evoked dACC-left dLPFC connectivity increased from pre-treatment to post-treatment (*t*(16)=2.753, *P*=0.014, *d*=0.668, 95% CI=[0.001; 1.337]). **d**, Task-evoked dACC-left dLPFC connectivity data individual patients, indicated by individual data points and connected by faint lines with a bolded line for sample mean. Error bars represent standard deviation. All statistical tests were two-sided and not adjusted for multiple comparisons. *Abbreviations:* dACC = dorsal anterior cingulate cortex; dLPFC = dorsolateral prefrontal cortex; GIR = guanfacine immediate release.

After 8 weeks of treatment with GIR, a general linear model with repeated measures, incorporating all 5 variables within the cognitive control circuit (3 activation measures and 2 connectivity measures), demonstrated a significant main effect of treatment (*F*(1,16) = 6.621, *P* = 0.020) on these measures. The interaction between the circuit variables and treatment was not significant. The main effect remained significant when including GIR dosage and the percentage of high motion volumes as covariates. Using planned contrasts, we demonstrated a significant increase toward the normative mean in both activation and connectivity within the cognitive control circuit, providing support for target engagement. The GIR effect on increasing circuit function reflected a medium effect size for both GoNoGo-evoked dACC activation (*t*(16) = 2.334, *P* = 0.033, *d* = 0.566, 95% CI=[0.021; 1.112], Fig. 3a) and connectivity between the dACC and left dLPFC (*t*(16) = 2.753, *P* = 0.014, *d* = 0.668, 95% CI=[0.001;1.337], Fig. 3c). Individual plots show that the majority of participants followed the same pattern (Figs. 3b and 3d). There was no significant change in right or left dLPFC activation or connectivity between the dACC and right dLPFC (**Supplementary Table 1**).

#### Specificity of cognitive control dysfunction and outcomes

In exploratory analyses, we evaluated the baseline mean of 31 regional circuit scores (activation or connectivity) across five circuits commonly implicated in subsets of depressed patients—attention, salience, default mode, positive affect, and negative affect. We aimed to identify patterns of neural deficits among individuals within the cognitive biotype + subgroup. Only the task-free attention circuit demonstrated connections with scores below − 0.5 standard deviations from the normative mean. We next evaluated the specificity of GIR’s effect by examining changes in these 31 regional circuit scores grouped by circuit using general linear models with repeated measures over the 8-week treatment course. No other circuit showed significant changes from pre- to post-treatment.

#### Cognitive behavioral performance outcomes

**Figure 4:**
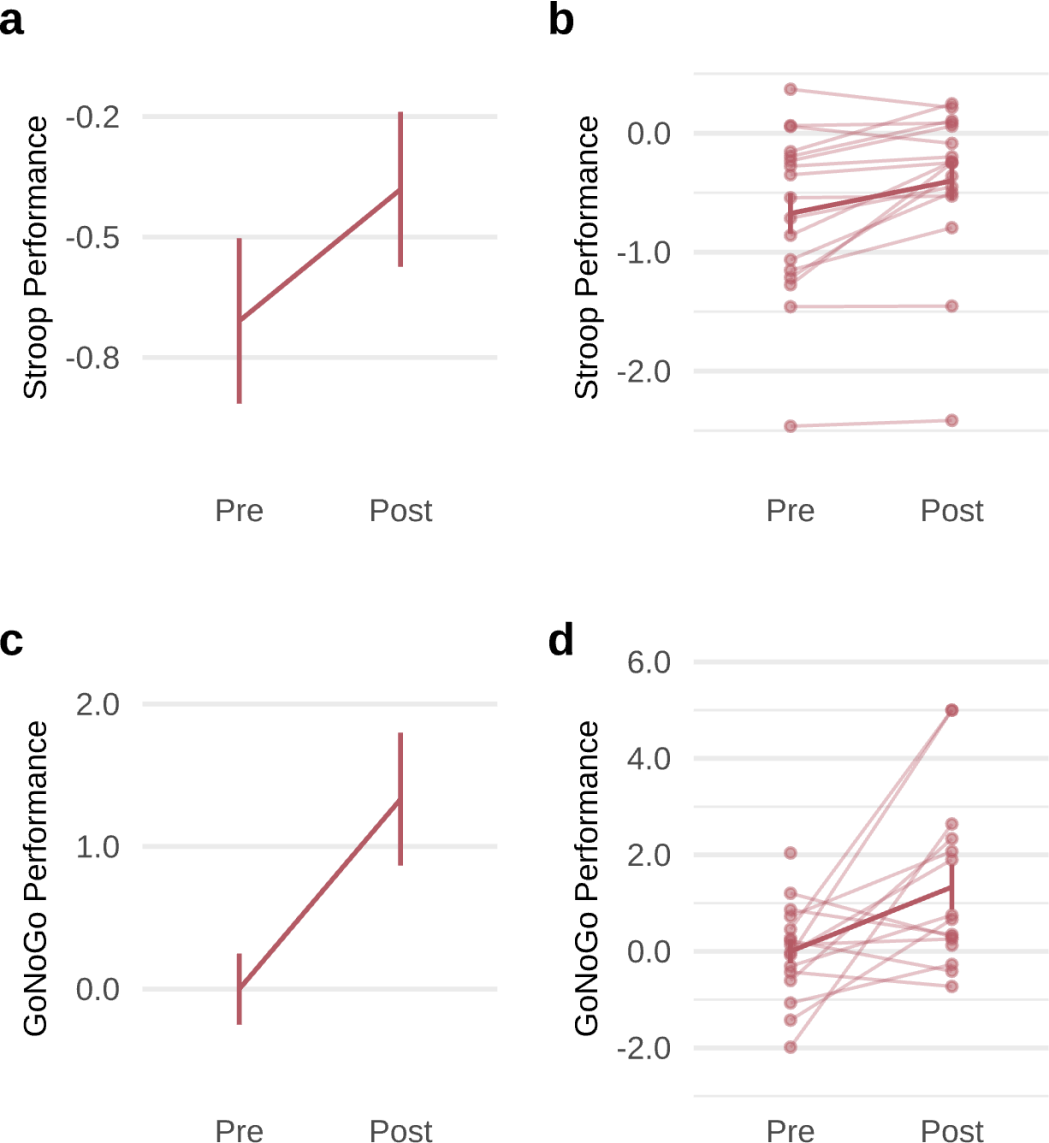
Improvements in cognitive behavioral performance, following 8 weeks of guanfacine immediate release. **a**, Cognitive behavioral performance was assessed using WebNeuro. Scores are expressed in z-scores, referenced to a healthy normative sample. Verbal interference (Stroop) performance improved from pre-treatment to post-treatment (*t*(16)=3.355, *P*=0.004, *d*=0.814, 95% CI=[0.564; 1.063]). **b**, Verbal interference (Stroop) performance data for individual patients, indicated by individual data points and connected by faint lines with a bolded line for sample mean. **c**, Performance on the GoNoGo task, measured by NoGo reaction time, improved from pre-treatment to post-treatment (*t*(16)=2.894, *P*=0.013, *d*=0.773, 95% CI=[0.047; 1.500]). **d**, GoNoGo performance data for individual patients, indicated by individual data points and connected by faint lines with a bolded line for sample mean.

After treatment with GIR, a general linear model with repeated measures including all 6 cognitive behavioral performance outcomes demonstrated a significant main effect of treatment (*F*(1,16) = 19.362, *P* < 0.001) as well as a significant interaction between these outcomes and treatment, indicating that the measures changed differentially with GIR (*F*(5,80) = 2.980, *P* = 0.016). We repeated this analysis with inclusion of GIR dosage and the main effect and interaction remained significant. When we ran planned contrasts, we observed significant improvements with large effect sizes on specific cognitive control performance measures relevant to our cognitive biotype. GIR-related improvements were observed for the capacity to selectively inhibit irrelevant information as measured by the Stroop Word task (*t*(16) = 3.355, *P* = 0.004, *d* = 0.814, 95% CI=[0.564; 1.063]; Fig. 4a) and NoGo reaction time (*t*(16) = 2.894, *P* = 0.013, *d* = 0.773, 95% CI=[0.047; 1.500]; Fig. 4c). The individual lines show broadly consistent effects across individuals for both tasks (Figs. 4b and 4d). No significant changes were found in the other cognitive behavioral measures (**Supplementary Table 1**).

#### Psychosocial outcomes

**Figure 5:**
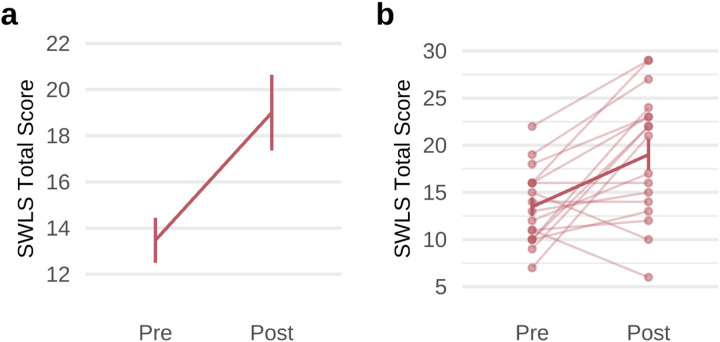
Improvements in global life satisfaction following 8 weeks of guanfacine immediate release. **a**, Global life satisfaction was measured with the Satisfaction With Life Scale (SWLS). This improved over the course of treatment (*t*(16)=3.633, *P*=0.002, *d*=0.881, 95% CI=[0.033; 0.640]). **b**, SWLS scores data for individual patients, indicated by individual data points and connected by faint lines with a bolded line for sample mean. Error bars represent standard deviation. All statistical tests were two-sided and not adjusted for multiple comparisons. *Abbreviation:* GIR = guanfacine immediate release.

After 8 weeks of treatment with GIR, a general linear model with repeated measures including all 4 variables within quality of life demonstrated a significant main effect of treatment (*F*(1,16) = 11.913, *P* = 0.003) and a significant interaction of treatment and quality of life (*F*(3,48) = 4.484, *P* = 0.007). A general linear model with repeated measures including GIR dosage as a covariate remained significant for the main effect and the interaction. Additionally, GIR treatment significantly improved quality of life across domains of physical health (*t*(16) = 3.159, *P* = 0.006, *d* = 0.881, 95% CI=[0.324; 1.438]; **Supplementary Fig. 1a**), psychological function (*t*(16) = 3.628, *P* = 0.002, *d* = 0.766, 95% CI=[0.319; 1.214]; **Supplementary Fig. 1b**), and social relationships (*t*(16) = 2.445, *P* = 0.026, *d* = 0.880, 95% CI=[0.288; 1.472]; **Supplementary Fig. 1c**) with moderate-to-large effect sizes. GIR also improved global satisfaction with life (*t*(16) = 3.633, *P* = 0.002, *d* = 0.881, 95% CI=[0.033; 0.640]; Fig. 5a–b) with a large effect size. We found no changes in the Environment domain or suicidal thoughts or behavior with GIR (**Supplementary Table 2**).

### Association between cognitive control circuit and secondary outcomes

**Figure 6:**
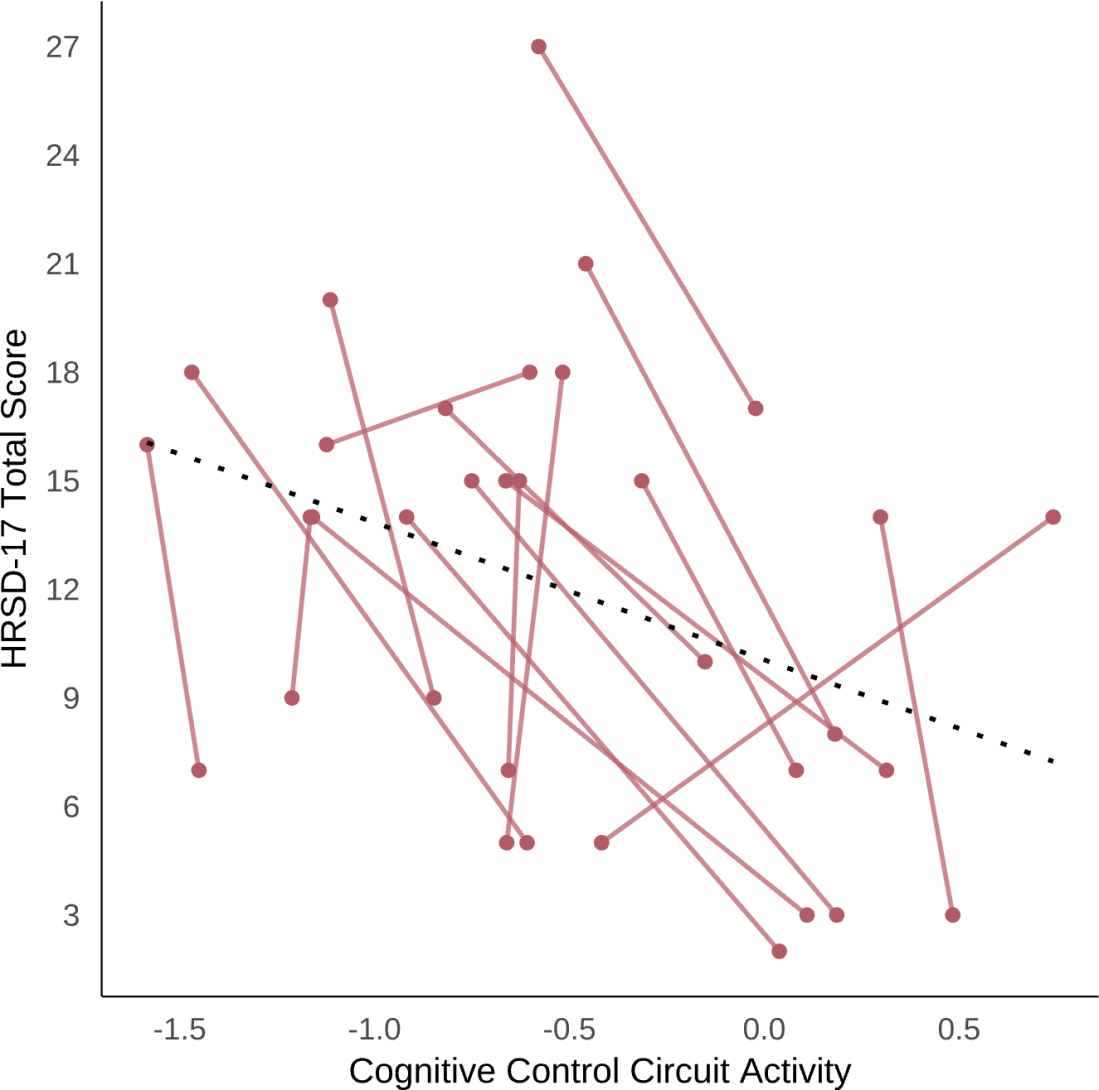
Association between cognitive control circuit connectivity and depression severity. Cognitive control circuit (dACC) activation is shown on the x-axis and HRSD-17 total score is shown on the y-axis. A repeated measures correlation was significant with r_rm_=−0.593, *P*=0.009. *Abbreviations:* HRSD-17 = 17-item Hamilton Rating Scale for Depression; dACC = dorsal anterior cingulate cortex.

Using repeated measures correlations, we found significant associations between changes over time in dACC activation and HRSD-17-assessed depression severity (r_rm_=−0.593, *P* = 0.009) (Fig. 6) and GoNoGo reaction time (r_rm_=0.566, *P* = 0.028). Furthermore, our results revealed significant associations between dACC-dLPFC functional connectivity and HRSD-17-assessed depression severity (r_rm_=−0.518, *P* = 0.028) and psychological health as assessed by the WHOQOL-BREF (r_rm_= 0.480, *P* = 0.044). These findings suggest the possibility that at least one mechanism through which depressive symptoms, cognitive behavioral performance, and function improve with selective α_2A_ agonism within the cognitive biotype + subgroup of depression is through modulation of the cognitive control circuit function.

## Discussion

We demonstrated that the selective α_2A_ agonist GIR significantly improved cognitive control circuit function, clinical measures, and cognitive control performance in patients with MDD who met criteria for the cognitive biotype + of depression. Specifically, after 8 weeks of treatment with GIR, patients showed increased activation in the dACC and enhanced connectivity between the dACC and left dLPFC during a cognitive control task, providing support for target engagement. Additionally, while GIR has been shown to have antidepressant properties in animals^16^, we demonstrated for the first time that GIR acts as an antidepressant in humans. GIR led to high rates of remission (64.7%) and response (76.5%), exceeding typical rates for standard antidepressants for the cognitive biotype of depression specifically^9^ as well as non-stratified samples of patients with MDD (e.g.,^22,23^). Behaviorally, GIR treatment resulted in improved performance on behavioral tests of cognitive control, both the GoNoGo and Stroop tests aspects of inhibition of irrelevant information and responses. These behavioral changes had moderate correlations with improved dACC activation, dACC – dLPFC connectivity, depression severity, physical health, psychological function, and social relationships, indicating convergence of GIR-induced improvements across circuit, behavioral and clinical domains. Furthermore, psychosocial function also significantly improved with treatment. Taken together, these findings are consistent with prior evidence that improvement in cognition is necessary for improvement in overall depression severity and psychosocial function within the cognitive biotype of depression^9^. Finally, we observed a positive, moderate strength association between an increase in cognitive control circuit activation and improvement in depression severity. This suggests the possibility that at least one mechanism through which depressive symptoms improve within the cognitive biotype of depression with selective α_2A_ agonism is modulation of cognitive control circuit function.

In this study, we used a composite biomarker that combined functional neuroimaging measures of cognitive control circuit function and behavioral measures of cognitive control performance from WebNeuro to define the cognitive biotype + subgroup of depression. WebNeuro measures alone provide an effective stratification filter for prospectively identifying the majority of participants with cognitive control circuit dysfunction (17/21 or 81%). However, the addition of the brain function measures in this composite biomarker may provide greater selectivity for identifying patients most likely to respond to GIR. For example, some individuals who meet WebNeuro criteria for cognitive control impairment may have deficits in other brain regions that are driving their cognitive impairment and are less relevant to the GIR mechanism of action.

Obtaining pre-treatment brain function measures allowed us to characterize the baseline neural dysfunction of the cognitive biotype + subgroup across five key circuits implicated in depression. In addition to deficits (by definition) in dLPFC activation within the cognitive control circuit, we found that our cohort had deficits in connections within the attention circuit (defined as < −0.5 s.d. below the normative mean) but not in any other circuit tested. This dysfunction in the task-free attention circuit likely reflects its close interaction with the task-evoked cognitive control circuit, as both networks collaborate to direct goal-oriented behavior. The absence of significant dysfunction in other circuits, including the task-free default mode and salience networks along with the task-evoked negative and positive affect circuits, suggests specificity in the neural abnormalities associated with the cognitive biotype + subgroup. This specificity underscores the importance of targeting interventions toward the identified neural dysfunctions, as a broader dysfunction across multiple networks would likely require a different therapeutic approach.

While the simultaneous collection of post-treatment symptom and brain circuit data limits our ability to draw definitive mechanistic conclusions, several findings strongly suggest that GIR improves depressive symptoms through a specific neural mechanism. Notably, by measuring activation and connectivity across six depression-related circuits, we demonstrated that GIR selectively targets the cognitive control circuit in individuals with dysfunction in this circuit, without affecting the other five circuits. In contrast, if changes had been observed across multiple circuits, we would have concluded that GIR likely exerts its effects through a broader, multi-network mechanism. Additionally, repeated measures correlations revealed significant relationships between cognitive control function, and improvement in secondary outcomes across symptom, cognitive behavioral, and function domains.

These promising results must be considered in the context of several limitations. Although the sample was prospectively selected using stringent criteria, it was relatively small. A larger sample size is needed to evaluate the generalizability of the findings and specific associations between changes in cognitive control circuit measures and clinical behavioral measures. Additionally, the BIG Study for Depression was an open-label trial. The use of a randomized controlled design would enable determination of the specificity of drug effects in circuit, behavioral, and clinical measures. Future randomized designs could also include participants who do not meet criteria for the cognitive biotype (i.e., a cognitive biotype-subgroup). While our results demonstrate the potential efficacy of GIR for the cognitive biotype, it remains unclear whether this targeted intervention would be similarly effective for patients without cognitive control deficits in circuit and behavior. It is possible that the beneficial effects of GIR on cognitive and clinical outcomes are specific to the cognitive biotype, consistent with the hypothesized mechanism of action targeting prefrontal dysfunction. Alternatively, GIR may have more general antidepressant effects that occur complementary to specific cognitive effects and extend beyond the cognitive biotype + subgroup of depression. The potential overlap of the cognitive biotype of depression with ADHD could also be explored in these studies. Furthermore, studies with a longer follow up could help determine whether GIR-induced changes in cognitive control circuit function predict longer-term clinical outcomes and whether early target engagement is necessary and sufficient for a sustained therapeutic response.

In this study, our focus was on the cognitive control circuit given its relevance to the cognitive biotype of depression. However, a promising future direction would be to examine GIR’s impact on other circuits relevant to depression. For example, GIR has been shown to regulate the cognitive control of emotion within the dLPFC and its connections to the amygdala. One small fMRI study showed that GIR selectively modulates coupling of the prefrontal cortex with the amygdala, altering the response bias for sad faces seen in depression^17^. Another promising future direction would be to examine the impact of GIR on inflammatory markers given that low-grade inflammation has been implicated as a pathological mechanism in about one-third of patients with depression^24^, particularly those with cognitive deficits^25^, and evidence indicates that GIR lowers inflammation through the deactivation of microglia^13^.

In conclusion, this study provides initial evidence for the promise of biomarker-stratified precision medicine approaches in depression. By using a composite behavioral and circuit biomarker to identify a subgroup of patients with depression and targeting the α_2A_ receptor with GIR, we demonstrate for the first improvements in neural circuit function, clinical outcomes, cognitive performance, and psychosocial outcomes. The outcomes highlight the advantages of selecting repurposed medications with known safety profiles for biomarker-stratified precision medicine trials and open the door for testing other selective α_2A_ compounds in the cognitive biotype + subgroup of depression. Furthermore, the results advance the objective to develop mechanistic experimental therapeutics for precision medicine in psychiatry, using biomarkers anchored in the Research Domain Criteria (RDoC) and focusing on patients underserved by current treatments.

## Methods

### Overview of the stratified precision medicine design

The BIG Study for Depression is a single-site, open-label, biomarker-stratified precision medicine trial investigating circuit, behavioral, and clinical endpoints in participants selected for the cognitive biotype of MDD and treated with the mechanistically selective treatment GIR. The study was registered on ClinicalTrials.gov, NCT04181736, and conducted at Stanford University School of Medicine, California. First enrollment was September 14, 2022, and final enrollment was October 27, 2023. All participants provided written informed consent after the procedures had been fully explained in accordance with Helsinki guidelines, under the approval of the Stanford University Institutional Review Board (#49147). See **Supplementary Fig. 3** for participant screening and recruitment details.

Participants completed stratification, baseline, and post-treatment assessment sessions. We analyzed data for 17 participants who met all per protocol criteria, including eligibility for the cognitive biotype + subgroup of MDD and who completed at least six weeks of GIR treatment.

Of these 17 participants, 16 (94.1%) completed the full eight weeks of treatment. We evaluated the effect of GIR on our primary fMRI circuit endpoints and secondary cognitive control performance, symptom, quality of life, and life satisfaction, and suicidality endpoints, comparing post-treatment to pre-treatment baseline sessions.

### Defining the cognitive biotype + subgroup of MDD

The cognitive biotype + subgroup was defined prospectively as required by our stratified precision medicine design. Prospective biotype stratification criteria encompassed clinical, behavioral, and circuit measures (Fig. 1). Clinically, each participant within the cognitive biotype + subgroup met criteria for moderate or greater depression severity indicated by a score of ≥ 14 on the HRSD-17. Behaviorally, each participant within this subgroup also had reduced performance on tests of cognitive control at a threshold of ≤ −0.5 s.d. below the healthy reference mean. Cognitive control circuit function in the dLPFC was assessed using functional magnetic resonance imaging and we confirmed using personalized circuit scores that each participant within the cognitive biotype + subgroup had reduced activation in this region, at a threshold of ≤ −0.5 s.d. below the healthy reference mean. These criteria and acquisition of the data to evaluate these criteria are described in greater detail in sections below. Our goal for circuit and behavioral criteria was to assigned individual participants to a biotype based on their relative extremes of performance and circuit function without assuming a specific statistical threshold and without under-sampling the biotype. We decided against a more extreme threshold, such as two standard deviations below the reference mean, as this would only capture approximately 2.5% of participants and we would risk under-sampling the biotype of interest.

### Study eligibility and prospective cognitive biotype confirmation

A pool of 150 participants were recruited directly from the community on the basis of initial study inclusion criteria: age between 18 and 69 years, self-reported symptoms of depression, fluent and literate in English, medication naïve to GIR, able to undergo a brain MRI and attend all study visits, and written informed consent. Further screening was undertaken in a series of steps to assess for general study inclusion and exclusion criteria, and for biotype specific criteria, listed in detail in **Supplementary Fig. 2**.

At the first step, we assessed biotype specific inclusion criteria for depressive symptom severity according to the 17-item Hamilton Rating Scale for Depression (HRSD-17)^26^ threshold of ≥ 14 undertaken by clinical coordinator personnel under the supervision of study clinicians. Participants also completed a psychiatric interview with clinical coordinators using the MINI-Plus structured interview^27^ in which the inclusion diagnosis of current, past, or recurrent nonpsychotic MDD according to DSM-5-TR criteria was assessed. The exclusion diagnoses of bipolar disorder, psychosis, obsessive compulsive disorder, post-traumatic stress disorder, attention deficit hyperactivation disorder, and substance use disorders and/or suicidal ideation representing imminent risk were also assessed. 80 participants met criteria at this step.

At the next step, participants completed the behavioral tests of cognitive control. Of the 80 participants who met HRSD-17 ≥ 14 and MINI-Plus criteria, we confirmed 52 also met our cognitive biotype + criteria for performance ≤ −0.5 s.d. below the healthy reference mean. These participants were then scheduled for a visit at the Stanford Clinical Translational Unit to assess medical eligibility for GIR.

Of these 52, 23 were excluded because of contraindications to GIR identified on medical history to exclude for any medical condition deemed unsafe, history of sudden cardiac death in a first degree relative, and/or use of a strong cytochrome P450 3A4 (CYP3A4) inhibitor; vital signs testing to exclude for hypotension (systolic blood pressure ≤ 90 and/or diastolic blood pressure ≤ 60 on 2 of 3 separate measurements at least 5 minutes apart) and/or bradycardia (≤ 55 beats per minute on 2 of 3 separate measurements at least 5 minutes apart); electrocardiogram to exclude for heart abnormalities; and/or lab tests to exclude for liver or kidney abnormalities deemed unsafe and/or positive drug screen for any substance deemed by the study physician to be unsafe for use with GIR in combination with other information obtained during screening.

At the next step, study personnel arranged for the 28 participants medically eligible for GIR to complete their pre-treatment baseline functional magnetic resonance imaging scan to confirm their circuit eligibility for the cognitive biotype + subgroup while they were medication free. Imaging details are provided under the baseline circuit assessment section below. In the majority of cases, personalized quantification of circuit scores, derived from baseline imaging, was confirmed prior to commencing GIR. Participants who had been taking psychotropic medications underwent a carefully monitored down-titration and subsequent washout period of five half-lives prior to commencing GIR under the direction of the participant’s primary mental health provider. In one case, due to clinical considerations, a participant continued on escitalopram 20 mg as an exception approved by the study team.

Circuit quantification confirmed that 24 of the 28 participants initiated on GIR met circuit criteria for the cognitive biotype + subgroup, defined by dLPFC activation ≤ −0.5 s.d. below the reference mean. Of the 4 excluded due to not meeting circuit criteria, it was deemed clinically necessary to commence treatment ahead of final circuit score confirmation.

Details of the treatment are provided in the GIR treatment section below. Of the 24 participants who met all cognitive biotype + criteria and were initiated on GIR, 17 completed at least 6 weeks of treatment and were defined as our per protocol participants for analysis. The other 7 did not complete due to side effects or other circumstances. Details of the participants included and excluded at each step in the BIG study flow are provided in the CONSORT chart in **Supplementary Fig. 2**.

### Per protocol cognitive biotype + sample characteristics

The resulting sample of 17 per protocol participants meeting all cognitive biotype + and treatment completion criteria had a mean age of 31.4 years [s.d.=11.1] and represented an equivalent number of females (47%) and males (53%). Within the sample, 29.4% were Caucasian, 41.2% were Asian, 11.8% were African American, 5.9% were of more than two races and the remaining 11.8% reported ‘other’ race.

Baseline depression severity assessed by the HRSD-17 was median 15 (range = 14–27), reflecting our clinical biotype criterion. Within this per protocol sample, 23.8% had a comorbid anxiety disorder as assessed by the Mini-International Neuropsychiatric Interview (MINI). The majority of participants (64.7%) had been treated for depression previously, and the median number of prior treatment failures was 2 0. The median final dose of GIR was 2mg nightly with a range of 0.25mg to 2mg. Further details about these sample characteristics are provided in **Supplementary Table 3**.

### GIR treatment

We selected GIR for this study because of its selectivity for α_2A_ receptor agonism, concentrated in the prefrontal circuit regions of interest for the cognitive biotype of depression. GIR’s selectivity for α_2A_ receptor agonism is 15–20x higher than for α_2B_ or α_2C_ receptors^28^. Steady state blood levels are attained within 4 days in most subjects. The average elimination half-life is approximately 17 hours (range 10–30 hours; FDA package insert^29^). Clinical studies have established the safety profile of GIR in humans. There is no evidence for tachyphylaxis with GIR in trials of adults for up to 8 weeks^30–32^. The most common adverse events include drowsiness, headache, fatigue, dizziness, and insomnia^33^.

For the participants in this study GIR was dosed once nightly to minimize the potential effects of sedation. GIR was commenced at a dose of 0.5mg and up titrated every 3 days by 0.5mg to a goal dose of 2 mg nightly by the second week of treatment. The total treatment period for the study was 8 weeks and participants met protocol criteria if they completed at least 6 weeks of treatment. Because of the open label design clinical personnel were not blinded to treatment status. However, all analyses were undertaken with personnel blind to the response status of individual participants. See supplementary text for further details of study treatment procedures.

### Adverse events

We assessed adverse events through verbal report during weekly virtual or in-person visits with either a trained, experienced research coordinator or a study clinician. Consistent with prior reports^33^, the most commonly reported adverse events were mild and included dry mouth (64%) and daytime fatigue (43%) as shown in **Supplementary Table 1**. Three participants withdrew from the study because of adverse events, two due to mild to moderate daytime fatigue, heart palpitations, dizziness, shortness of breath, and difficulty sleeping, and one due to a serious adverse event, worsening of suicidal ideation. This participant was withdrawn from the study.

### Study assessments and assessment sessions

All steps in the study flow are outlined in **Supplementary Fig. 4**.

1. A *prospective screening session* to confirm clinical and cognitive behavioral criteria for stratification of the cognitive biotype + subgroup. This session was embedded within the multi-step process for assessing participant eligibility, outlined in the above Methods sections. During this session, the HRSD-17 was used to identify those participants who met our clinical criteria of moderate or greater depression severity (HRSD-17 ≥ 14) for the cognitive biotype + subgroup. Participants were also provided with access to the WebNeuro cognitive behavioral tests which they could complete on their own laptop.
2. A pre-treatment baseline session to confirm circuit criteria for the cognitive biotype + subgroup and to establish baseline activation for primary circuit measures, as well as secondary measures of cognitive behavior, symptoms, and functioning. During this pre-treatment session, we re-assessed participants on the HRSD-17 and WebNeuro cognitive behavioral tests to establish the pre-treatment baseline for these measures. Participants also underwent functional magnetic resonance imaging scans to assess our primary measures of dLPFC and dACC activation and connectivity within the cognitive control circuit. Additional secondary measures of symptoms and function were also assessed at baseline.
3. A post-treatment session to assess the effect of GIR on primary circuit measures, as well as secondary measures of cognitive behavior, symptoms, and functioning. During the post-treatment session, we re-assessed participants on each of the primary and secondary measures after 8 weeks of treatment with GIR.

The primary and secondary measures assessed in each of these sessions are detailed in the following assessment sub-sections.

### Cognitive control circuit measures

Cognitive control circuit activation was our primary measure for the BIG study. Circuit criteria for the cognitive biotype + subgroup were verified during the pre-treatment baseline scan. Circuit measures were derived from fMRI data acquired during a cognitive control GoNoGo task, preprocessed and quantified using the established Stanford EtCere Image Processing pipeline implemented in a singularity environment in C + + to ensure reproducibility across computational systems^34,35^. This procedure enabled us to determine which patients met criteria for dLPFC activation ≤ −0.5 s.d. below the healthy reference mean. The same quantification approach was used at the post-treatment session, enabling us to evaluate GIR’s effect on change in cognitive control circuit function in standardized units.

### Acquisition

fMRI data were acquired at the Stanford Center for Cognitive Neurobiological Imaging using a GE 3 Tesla UHP scanner (GE Healthcare, Milwaukee, WI, USA) with a Nova Medical 32-channel head coil. Head motion was restricted with foam pads and participant alertness was monitored using an eye-tracking system. Head motion was also recorded, which was later subject to quality control and potential data exclusion on the premise of excess motion. An established protocol was used: TR = 2s, TE = 30ms, flip angle = 54°, FOV = 220.8×220.8mm, 92×92 matrix, 60 slices, 2.4mm thickness, calibration volumes = 2. We also acquired a high-resolution T1-weighted MRI sequence for anatomical normalization. A total of 180 contiguous slices, each 1 mm thick, covered the whole brain with an in-plane resolution of 1mm × 1mm. It was acquired in the sagittal plane using a 3D spoiled gradient echo sequence (TR = 8.3ms; TE = 3.2ms; flip angle = 11 degrees; TI = 500ms, NEX = 1 and ASSSET = 1.5; frequency direction: S/I; matrix = 256 × 256, 180 contiguous slices, 1mm isotropic voxels).

During scanning, participants completed the GoNoGo task^36,37^. which probes the cognitive control circuit. The GoNoGo task was presented using the acquisition component of the Stanford EtCere Image Processing System protocol. In this task,’Go’ trials (the word “press” in GREEN) required participants to respond as quickly as possible, while the ‘NoGo’ trials (“press” in RED) required participants to withhold responses. 180 Go and 60 NoGo stimuli were presented in pseudorandom order; stimulus duration was 500 ms each with an interstimulus interval of 750 ms^38^.

Post-acquisition, we generated task-evoked circuit scores for the cognitive control circuit using the quantification component of the established Stanford EtCere Image Processing pipeline^34,35^. We previously demonstrated the reliability, validity, and generalizability of this system, as well as sensitivity to individual for clinical participants^34,35^. A summary of the steps involved in quantification, including preprocessing, extraction of regions of interest for the cognitive control circuit, analysis of task-evoked activation within these regions and standardization of activation relative to our reference dataset is provided in the following sub-sections.

### Preprocessing

All scans were reviewed to be free of incidental findings, major scanner artifacts such as signal dropout. The first three volumes of the functional acquisition were removed to ensure that the images quantified for analysis had reached steady state. Motion correction was implemented with scripts that use well established procedure to realign and unwarp functional images to the first image of each task run. T1-weighted images were normalized to Montreal Neurological Institute space using nonlinear registration, and functional images were co-registered to the these T1-weighted images using procedures based on the Functional Magnetic Resonance Imaging of the Brain (FMRIB) Software Library (FSL)^39^. Co-registered images were high-pass filtered using a cutoff period of 128 seconds. Data for all participants met our prior established quality control ‘pass’ criteria for head motion, which is a threshold of less than 25% of time points censored for frame-wise displacement, and for adequate temporal signal to noise ratio, which is a threshold of 50 or greater.

### Derivation of regions of interest defining the cognitive control circuit

The Stanford EtCere Image Processing System uses dLPFC and dACC regions of interest to define the cognitive control circuit and these regions defined using the meta-analytic platform Neurosynth^40^ and meet prior established quality control and psychometric criteria^34,35^.

### Task-evoked activation analysis

The task-evoked analysis was conducted using the ‘Stanford Et Cere Image Processing System’ in C++. Functional images were registered to the structural images. Task-evoked activation was quantified using a generalized linear model analysis in which the order of ‘NoGo’ and ‘Go’ stimuli were recorded as task events and convolved with a canonical hemodynamic response. Activation in dLPFC and dACC were quantified as beta estimates and expressed in standard deviation units relative to the mean and standard deviation of a healthy reference dataset, which provided a standard benchmark for interpreting a score for each individual patient. GoNoGo task-related connectivity between dLPFC and dACC was quantified using a psychophysiological interaction (PPI) method. PPI was used to seed connectivity from dACC to left and right dLPFC, and from left and right dLPFC to ACC, and we then averaged the two directions of connectivity for each pair of regions. This approach to quantifying clinical participant-level data has been validated in our prior work^34^.

### Norm-referencing of individual circuit scores

Each of the activation and functional connectivity measures for the dLPFC and dACC regions defining the cognitive control circuit were expressed in standard deviation units relative to the mean and standard deviation of our healthy reference dataset, acquired on the same scanner. This standardized referencing method enabled the personalized interpretation of circuit scores for each individual participant. Specifically, this procedure enabled us to determine which patients met criteria for GoNoGo-evoked circuit scores ≤−0.5 s.d. below the healthy reference mean, and to evaluate change cognitive control circuit function in standard deviations induced by treatment with GIR.

### Cognitive behavioral performance

Cognitive behavioral performance was a secondary measure for the BIG study. Cognitive behavioral performance criteria for the cognitive biotype + subgroup were verified as part of our prospective stratification procedure during participant screening. Stratification criteria for the cognitive biotype + subgroup were established using cutoffs on behavioral tests of cognitive control, including Maze task, GoNoGo, Digit Span, and Verbal Interference tests. The cutoff for performance was ≤−0.5 s.d. below the normative mean on one or more of these cognitive control tests. These tests were selected to assess complementary aspects of cognitive control that implicate dorsal prefrontal brain regions. We drew on a cognitive neuroscience-based framework for cognitive control, emphasizing goal-directed action selection and response inhibition^41^, along with classical cognitive theory, which includes working memory, interference suppression^42,43^.

The same cognitive behavioral tests were re-administered at the pre-treatment baseline scan and at the post-treatment session. Parallel forms of the tests were used to minimize practice effects. Thus, we selected the Maze test to assess goal selection, GoNoGo to assess response inhibition, Digit Span to assess working memory, a Verbal Interference test (analogous to the Stroop) to assess suppression of interfering information. At baseline and post-treatment, we also included a Switching of Attention Test (analogous to Trails B) to assess processing speed which is implicated in some classical theories of cognitive control^42,43^.

Performance on each test was represented by a composite score expressed as a standard deviation score referenced to a healthy norm reference group. Where needed, extreme scores were winsorized to a threshold of 5 standard deviations. Composite scores were obtained by averaging performance on each individual test score: trials completed, completion time, path learning time, total errors, and overrun errors from the Maze, maximum recall span and correct trials from Digit Span, total errors and reaction time for Verbal Interference, and completion time, average connection time, and errors in the Switching of Attention Test. Because GoNoGo data was obtained from in-scanner, only reaction times were used.

Behavioral performance was on these tests was assessed using the standardized, computerized test battery, WebNeuro^44^, which has established norms across nine decades of the healthy lifespan^45–47^, test-retest reliability over an 8-week re-test period relevant to this study^48^, construct validity with respect to traditional neuropsychological batteries and brain measures^49,50^, and utility in distinguishing cognitive impairments in psychiatric groups^51–54^. Cross-cultural consistency has also been established^55^.

### Symptom assessments

#### Depression symptom severity

Observer rated depression symptom severity was a secondary clinical measure for the study. Moderate or greater depression symptom severity for the cognitive biotype + subgroup was verified as part of our prospective stratification procedure during participant screening using the HRSD-17, with a threshold of the criterion for moderate or greater severity ≥ 14, as outlined in the above study eligibility section. Within the HRSD-17, items related to mood, guilt, suicide, energy and interest, psychomotor slowing or agitation, anxiety, and bodily preoccupation are rated on a 5-point scale, items related to weight on a 4-point scale, and items related to sleep, somatic symptoms, and insight on a 3-point scale. The HRSD-17 scores range from 0 (no depression) to 52 (severe depression).

The HRSD-17 was re-administered at the pre-treatment baseline scan and at the post-treatment session. At post-treatment we assessed the continuous change in depression severity. We also evaluated categorical outcomes of response defined as a percent symptom improvement of ≥ 50% from baseline on the HRSD-17 and remission of depression defined as a score of ≤ 7 on the HRSD-17.

#### Suicidality

Suicidality was assessed using the observer-rated 5-item ideation subscale of the Columbia-Suicide Severity Rating Scale (C-SSRS)^56^ as an additional secondary clinical measure. Scores range from 0 (no suicidal ideation) to 5 (high suicidal ideation). The C-SSRS was administered at the pre-treatment baseline and post-treatment but was not used prospectively to define the cognitive biotype + subgroup.

### Psychosocial assessments

In addition to symptom measures, we also included secondary evaluations of function at the pre-treatment baseline and the post-treatment follow-up sessions. The World Health Organization Quality of Life – Brief (WHOQOL-BREF) Scale, a 26-item questionnaire was used to assess quality of life in the domains of physical health, psychological function, social relationships, and environment^57^. All questions contain 5 items and the scores for each domain are transformed to a scale of 0–100. Global life satisfaction was assessed using the Satisfaction With Life Scale, a 5-item questionnaire that measures global cognitive judgments of satisfaction with an individual’s life using a 7-item Likert scale^58,59^. The scores range from 5–35.

### Outcome measures after 8 weeks of treatment with GIR

To assess the effect of GIR we evaluated the pre- versus post-treatment change in our primary mechanistic outcome measure of cognitive control circuit function, and in our secondary outcomes of cognitive behavioral performance, depressive symptom severity (including response and remission), psychosocial function, and suicidality.

### Statistical analysis

All statistical analyses were conducted in R studio version 2022.12.0 + 353. For our primary cognitive control circuit measure we tested the effect of GIR using a general linear model including a within subjects’ effect for treatment (pre- versus post-treatment sessions) and for circuit function, with five repeated measures for each circuit measure defining the cognitive control circuit. We tested for the main effect of treatment and, secondarily, an interaction between treatment and circuit measure. Planned paired t-test contrasts tested for the effect of GIR on change in these individual circuit measures.

For secondary measures, general linear models were also undertaken with treatment as a within-subjects factor and a within-subjects repeated measures factor for assessments in which there were multiple observations. Cognitive behavioral performance comprised 6 test measures of cognitive control and, quality of life, four domains. Planned paired t-test contrasts tested for the effect of GIR on change in each set of individual secondary measures in each general linear model. The secondary measures of depression symptom severity and suicidality were exceptions that did not have repeated measures.

A two-sided significance level of 0.05 was set for each analysis within each modality for our primary and secondary outcomes. We did not correct for the number of analyses across modalities. Each outcome was included to test a specific hypothesis about the effect of GIR on circuit, behavioral, symptom, and functional aspects of cognitive control. In addition to statistical significance, to aid interpretation of clinical meaningfulness, we reported effect sizes for the change in each outcome measure. Change from baseline to post-treatment effect sizes were computed as Cohen’s d for paired t-tests. For primary circuit and secondary outcomes that showed a significant effect of GIR, we evaluated the association between these primary and secondary outcomes using repeated measures correlations. Finally, we reran general linear models with repeated measures to evaluate if effects of GIR were related to relevant covariates including GIR dosage and individual variations in head motion while in the scanner. In exploratory analyses, we assessed the specificity of dysfunction in the cognitive biotype + subgroup at baseline as well as the specificity of change by examining regional circuit scores across five circuits commonly implicated in subsets of depressed patients—attention, salience, default mode, positive affect, and negative affect.

## Data Availability

Data collected for the study, including individual participant data and a data dictionary defining each field in the set, will be made available after approval of a proposal with investigator support.
